# New Insights of Emerging SARS-CoV-2: Epidemiology, Etiology, Clinical Features, Clinical Treatment, and Prevention

**DOI:** 10.3389/fcell.2020.00410

**Published:** 2020-05-22

**Authors:** Gangqiang Guo, Lele Ye, Kan Pan, Yu Chen, Dong Xing, Kejing Yan, Zhiyuan Chen, Ning Ding, Wenshu Li, Hong Huang, Lifang Zhang, Xiaokun Li, Xiangyang Xue

**Affiliations:** ^1^Department of Microbiology and Immunology, School of Basic Medical Sciences, Institute of Molecular Virology and Immunology, Institute of Tropical Medicine, Wenzhou Medical University, Wenzhou, China; ^2^Department of Gynecologic Oncology, Wenzhou Central Hospital, Wenzhou, China; ^3^First Clinical College, Wenzhou Medical University, Wenzhou, China; ^4^Second Clinical College, Wenzhou Medical University, Wenzhou, China; ^5^Center for Health Assessment, Wenzhou Medical University, Wenzhou, China; ^6^School of Pharmaceutical Sciences, Wenzhou Medical University, Wenzhou, China; ^7^Institute of Virology, Wenzhou Medical University, Wenzhou, China

**Keywords:** SARS-CoV-2, coronavirus, epidemiology, etiology, clinical features, clinical treatment and prevention

## Abstract

Since the first reports that the novel coronavirus was showing human-to-human transmission characteristics and asymptomatic cases, the number of patients with associated pneumonia has continued to rise and the epidemic has grown. It now threatens the health and lives of people across the world. The governments of many countries have attached great importance to the prevention of SARS-CoV-2, via research into the etiology and epidemiology of this newly emerged disease. Clinical signs, treatment, and prevention characteristics of the novel coronavirus pneumonia have been receiving attention worldwide, especially from medical personnel. However, owing to the different experimental methods, sample sizes, sample sources, and research perspectives of various studies, results have been inconsistent, or relate to an isolated aspect of the virus or the disease it causes. Currently, systematic summary data on the novel coronavirus are limited. This review combines experimental and clinical evidence into a systematic analysis and summary of the current progress of research into SARS-CoV-2, from multiple perspectives, with the aim of gaining a better overall understanding of the disease. Our report provides important information for current clinicians, for the prevention and treatment of COVID-19 pneumonia.

## Introduction

Severe acute respiratory syndrome coronavirus 2 (SARS-CoV-2) is a novel, zoonotic, positive-sense, single-stranded RNA betacoronavirus (sub-genus *Sarbecovirus*, sub-family *Orthocoronaviridae*). This sub-family also includes SARS-CoV and MERS-CoV (Middle Eastern respiratory syndrome), and the SARS-like (SL) viruses of bats: bat-SL-CoVZC45 and bat-SL-CoVZXC21 (Chan et al., 2020). Coronavirus disease 2019 (COVID-19), the novel coronavirus pneumonia, was caused by SARS-CoV-2. On January 30, 2020, the World Health Organization (WHO) declared it a Public Health Emergency of International Concern, and on February 28 it raised the global risk of COVID-19 to the highest level. On March 11, a global pandemic was declared. Given the rapid global spread of SARS-CoV-2, there is an urgent need for large-sample data analyses and clinical research of cases in worldwide. This would improve the accuracy of our understanding of the epidemiology and clinical characteristics of SARS-CoV-2 and might also reveal pathogenic mechanisms and potential risk factors. A large number of studies and case reports have begun to answer these questions, but there is a lack of systematic analysis and summation.

This study summarizes the clinical data of patients infected with SARS-CoV-2, as of April 29, 2020, the research results reported so far, the detailed epidemiological, clinical, etiological, and immunological characteristics of SARS-CoV-2, and advances in drugs for prevention and treatment, which provide a basis for formulating more accurate medical treatment strategies. The emergence and large-scale outbreaks of SARS-CoV, MERS-CoV, and now SARS-CoV-2 remind us that infectious diseases caused by coronaviruses are a serious, global health threat. With changes in global climate and ecological environments, and increased opportunities for human-animal contact, it is probable that mutated, novel coronaviruses will continue to appear in the future, with harmful consequences to human health. This article systematically analyzes our knowledge of COVID-19 (caused by SARS-CoV-2) from multiple perspectives, in the hope of helping others to formulate scientific prevention and treatment strategies, both now and in the future.

## Epidemiological Characteristics

### Changes in Infection and Mortality Rates of Confirmed and Suspected Cases

**Figure 1:**
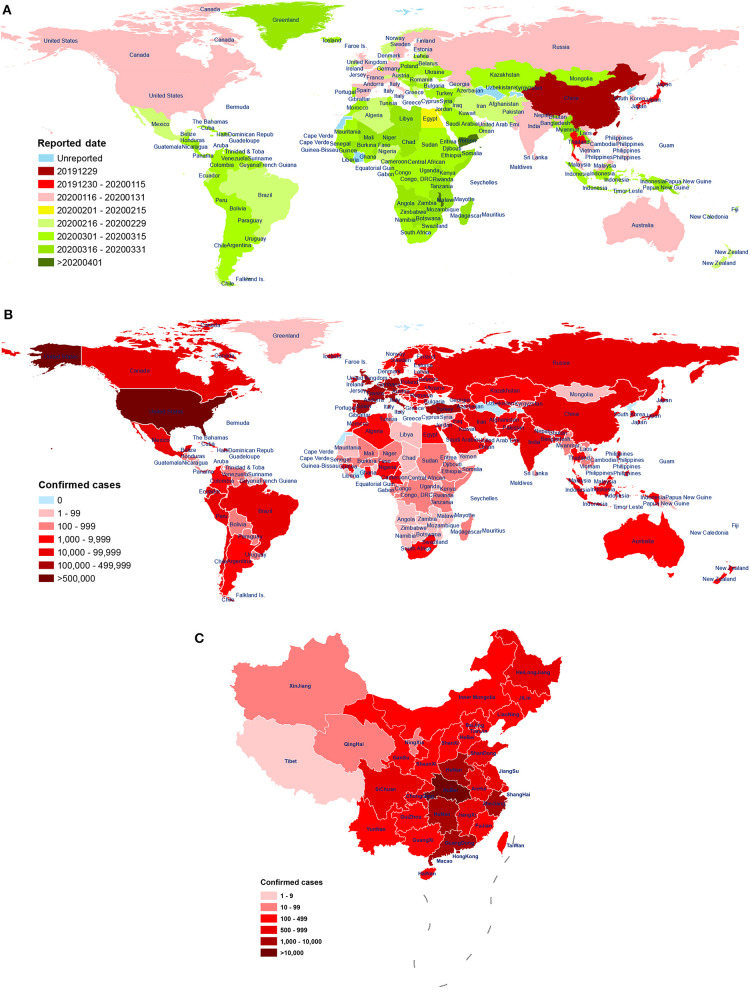
The distribution of patients across world. **(A)** First reported date of case, by country, throughout world, as of 28 April 2020. The date of the first reported COVID-19 patient in 213 countries and regions around the world. The time sequence of reporting for each country is labeled according to the earliest (red) and latest (green) date of onset. Blue indicates no reporting available. Data source: World Health Organization (WHO); **(B)** The distribution of laboratory-confirmed cases throughout world, as of 28 April 2020. Spatial distribution of the 2,954,222 cases of COVID-19 diagnosed around the world. The cumulative number of confirmed diagnoses in each country is labeled in shades of red. Blue indicates no confirmed cases. Data source: People's Daily, Chinese Center for Disease Control and Prevention; World Health Organization (WHO); **(C)** The distribution of laboratory-confirmed cases throughout China, as of 29 April 2020. Distribution of the 84,369 cases of COVID-19 were diagnosed in China (including Hong Kong, Macao, and Taiwan) by city. The cumulative number of confirmed diagnoses in each city is labeled in shades of red. Data source: Chinese Center for Disease Control and Prevention.

The virus has been reported in many countries (including the United States, Spain, Italy, the United Kingdom, Germany, France, and Turkey; Figure 1A). As of April 29, 2020, there have been a total of 2,954,222 diagnosed cases of novel coronavirus pneumonia and 202,597 deaths (mortality rate: 6.86%) in 213 regions or countries worldwide. Of these cases, China (including Hong Kong, Macao, and Taiwan) has reported a total of 84,369 confirmed cases, 7 suspected cases, and 4,643 deaths (mortality rate: 5.50%). The number of diagnoses and deaths continues to increase worldwide and poses a continuing, growing health threat (Figure 1B). These data show that the number of patients infected with SARS-CoV-2 is already much higher than the number infected by the emergence of SARS-CoV in 2002-03 (8,098) and MERS-CoV in 2012 (2,254), suggesting a higher rate of infection per exposure. Encouragingly, the number of confirmed cases in most cities in China has been declining over time. However, confirmed cases have started to appear in countries such as Vietnam (Phan et al., 2020) and Nepal (Bastola et al., 2020), both primary and secondary infections have been found in South Korea (Ki and Task Force for 2019-nCoV, 2020), and the epidemic has continued to worsen in countries such as the United States, Spain, Italy, the United Kingdom, and Germany.

One study reported a mortality rate of 15% (6 cases) among 41 COVID-19 patients in Wuhan, China, as of January 2 (Huang et al., 2020). Another study reported a mortality rate of 11% (11 deaths) among 99 COVID-19 patients in Wuhan, as of January 25 (Chen N. et al., 2020). Wang et al. reported a mortality rate of 4.3% (6 deaths) among 138 hospitalized COVID-19 patients in Wuhan, as of February 3 (Wang D. et al., 2020). As of February 29, 2020, mortality rate of symptomatic COVID-19 patients in Wuhan was 1.4% (0.9–2.1%), which was lower than 3.4% determined by the World Health Organization (Wu J. T. et al., 2020). Over time, the case fatality rate has fallen greatly, which may be due to the improvement in hospital treatment methods, and inconsistency in the severity of disease among infected patients in different analysis groups. Moreover, diagnostic and detection bias might also be one of the reasons for the potential regional differences. A study of samples from across China found a mortality rate of 3.06% (95% CI: 2.02–4.59%) among 8,866 cases, as of January 26 (Yang et al., 2020). Subsequently, Guan et al. reported a mortality rate of 1.36% (15 deaths) among 1,099 COVID-19 patients, as of January 29 (Guan et al., 2020a). Guan et al. further reported a case fatality rate of 2.3% (1,023 cases) and a nationwide (excluding Hubei) case fatality rate of only 0.39% from 72,314 cases, as of February 11; The crude case fatality rate in Hubei Province (2.9%) was 7.3 times higher than that in other provinces (0.4%), indicating that COVID-19 patient deaths in China were mainly concentrated in the Hubei Province (Novel Coronavirus Pneumonia Emergency Response Epidemiology Team, 2020) (Figure 1C). In summary, the study of samples from across China showed that the mortality rate of COVID-19 patients was lower than that of other coronavirus epidemics, including severe acute respiratory syndrome (SARS, 40% mortality rate for ages 60 and above) (Donnelly et al., 2003) and Middle East respiratory syndrome (MERS, 30% mortality rate) (Ahmed, 2017). In addition, compared to the case fatality rate of COVID-19 patients in Wuhan, Hubei, the overall rate in China is significantly lower. As recent studies have shown, temperature variation and humidity, cellular immune function, age interval, medical service, comorbidities, and gender may be important factors affecting the COVID-19 mortality (Caramelo et al., 2020; Jin J.-M. et al., 2020; Li H. et al., 2020; Ma Y. et al., 2020; Zeng Q. et al., 2020). Moreover, it should be noted that the number of confirmed and suspected cases is still increasing worldwide. Of the 393 initial patients with COVID-19 admitted in New York City, 40 (10.2%) have died, and 260 (66.2%) have been discharged from the hospital, as of April 10th (Goyal et al., 2020). Therefore, with the continuing spread of the pandemic and the inclusion of more regional samples, the infection and mortality rates of the disease continue to change, and secondary and higher-order patients have appeared.

### Controversy Regarding Sources of Infection

Zhou et al. found, through next-generation metagenomic sequencing and real-time PCR analysis, that SARS-CoV-2, which causes COVID-19, may have originated from bats (Zhou et al., 2020). Wu et al. found that COVID-19 is caused by a novel coronavirus with a sequence highly similar to that of SARS-CoV (Wu F. et al., 2020). This coronavirus has mainly been found in the *Rhinolophus sinicus*, and similarly caused a large-scale infectious outbreak, first reported in Asia in 2003 (de Wit et al., 2016; Yin and Wunderink, 2018; Cui et al., 2019; Song et al., 2019). The evidence suggests that SARS-CoV-2 may also originate from the *Rhinolophus sinicus*. A recent study by Guo Q. et al. (2020) compared coronavirus infection patterns among vertebrate hosts and found that mink coronavirus, rather than bat coronavirus, shows a closer infection pattern to SARS-CoV-2, suggesting that mink may be an intermediate host for SARS-CoV-2. Another study identified snakes as the most likely source of infection, by analysis of synonymous codon usage bias (Ji et al., 2020). Liu et al. found, through metagenomic sequencing analysis, that SARS-CoV is the most widely distributed among the coronaviruses detected in the Malayan pangolin (*Manis javanica*) (Liu P. et al., 2019). Further, whole genome sequencing and lineage analysis by South China Agricultural University found that pangolins may be potential intermediate hosts of SARS-CoV-2 (Lam et al., 2020). Molecular and phylogenetic analysis by Liu et al. showed that, although pangolin coronavirus is genetically related to SARS-CoV-2 and bat coronavirus, direct descent of SARS-CoV-2 from pangolin coronavirus is not supported, and they suggest that the pangolin is unlikely to be an intermediate host for SARS-CoV-2 (Liu P. et al., 2020). Most species of bats inhabit tropical and subtropical rain forests or caves and roost far from areas of human activity. Viruses from bats need to enter animal hosts, such as mink, snakes, and pangolins, that have closer contact with humans, to continue their evolution, and may become able to spread to humans after some degree of mutation and recombination (Paules et al., 2020). SARS-CoV-2 may have one or more intermediate hosts between wild animals and humans.

Yu et al. found, through genetic analysis, that SARS-CoV-2 may have been imported into the Huanan Seafood Market from elsewhere (Yu W.-B. et al., 2020). Some research's reported that there were many early patients who had no history of exposure to the Huanan Seafood Market in Wuhan (Chen N. et al., 2020; Huang et al., 2020). Moreover, another research reported that three central variants distinguished by amino acid changes were found, which were named A, B, and C, with A being the ancestral type according to the bat outgroup coronavirus. The A and C types are found in significant proportions outside East Asia, that is, in Europeans and Americans (Forster et al., 2020). Taken together, these results indicated that China is not the virus or the disease origin. The current method of spread has changed from zoonotic to human-to-human transmission, and asymptomatic infected persons also have the potential to spread the disease (General Office of National Health Commission, 2020b), which may become a key point of epidemic control. Dong et al., reported that 55.4% of the 2,135 confirmed or suspected children had mild or no symptoms (Dong et al., 2020). The results of census of 215 pregnant women infected with SARS-CoV-2 showed that the number of asymptomatic infections was 7.25 times higher than that of COVID-19 (Sutton et al., 2020). An asymptomatic infected person releases the same amount of virus as a patient with symptoms. It is suggested that asymptomatic infection is highly contagious, but the specific severity is still unclear (Zou L. et al., 2020). Thirty to Sixty percentage of people infected with SARS-CoV-2 are asymptomatic or mild, but still have the ability to spread the virus, which may trigger a new rounds of outbreak (Qiu, 2020). In summary, it is important to confirm the source and intermediate hosts of the virus, as soon as possible. Not only will this allow prevention of further zoonotic transmission, but it can also assist in the development of drugs and vaccines against the virus.

### Routes of Transmission

The main route of SARS-CoV-2 transmission is through respiratory droplets and close contact. In a relatively closed environment, there is a possibility of aerosol transmission when exposed to high concentrations of aerosol for a long period of time. Other routes, such as fecal-oral, mother-to-child, urine, and bloodborne transmission need to be confirmed by further research. (1) Droplet transmission: COVID-19 patients produce droplets which temporarily stay in the air within a radius of 4 m, through coughing, sneezing, talking, and so on. This can cause infections in vulnerable persons, after inhalation (General Office of National Health Commission, 2020b; Jiang et al., 2020; Lu et al., 2020); (2) Contact transmission: Droplets containing SARS-CoV-2 are deposited on the surface of objects. After the hands of vulnerable persons become contaminated by contact, they can then be moved to the mucous membranes of the oral cavity, nasal cavity, eyes, and so on, and cause infection (General Office of National Health Commission, 2020b; Rothe et al., 2020); (3) Fecal-oral transmission: in multiple locations, SARS-CoV-2 has been detected in the esophagus, gastrointestinal tract, and feces of confirmed patients (Pan et al., 2020), indicating that the virus can replicate and survive in the digestive tract and suggesting a possible risk of fecal-oral transmission (Gimeno et al., 2008; Commission, 2020; Guan et al., 2020a); (4) Mother-to-child transmission: SARS-CoV and MERS-CoV can cause serious complications during pregnancy (Wong C. K. et al., 2004; Alfaraj et al., 2019), and the similar pathogenicity and high degree of sequence homology between SARS-CoV-2, SARS-CoV, and MERS-CoV (Mahase, 2020) suggests that SARS-CoV-2 may also cause severe maternal and/or perinatal complications (Huang et al., 2020). However, none of the 9 pregnant women infected with SARS-CoV-2 and treated at Zhongnan Hospital of Wuhan University progressed to severe pneumonia, and SARS-CoV-2 test results of amniotic fluid, umbilical cord blood, breast milk samples, and neonatal throat swab samples were all negative (Chen H. et al., 2020), indicating that there is no evidence that SARS-CoV-2 can cause serious adverse consequences in the newborn or spread to the fetus in the womb. Similarly, there is also no evidence of perinatal SARS infection among infants born to these mothers (Wong S. F. et al., 2004). There have been recent reports of cases of SARS-CoV-2 infection in women confirmed to be pregnant (Zeng L. et al., 2020), indicating a significant possibility of mother-to-child transmission, but the possibility of exposure to infection at birth cannot be ruled out. Due to limited sample size, the gestational age, and the incomplete state of sample collection, it is not completely clear whether SARS-CoV-2 is transmitted from mother to child; (5) Other routes of transmission: in COVID-19 patients with conjunctivitis, SARS-CoV-2 was detected in tears and conjunctival secretions (Xia et al., 2020b). Rhesus macaques can be effectively infected with SARS-CoV-2 via ocular conjunctival route (Deng et al., 2020). Zhong et al. also isolated novel coronavirus from a urine sample of a COVID-19 patient. Thus, these must also be considered as possible routes of transmission, via environmental contamination. Clarifying the specific types of transmission route helps to protect healthy people, and thus reduces the infection rate in the population.

### Vulnerable Populations

As an emerging infectious disease, the whole population is broadly vulnerable to COVID-19. However, most patients have been between the age of 30 and 69 years (44,672 cases, 77.8%) (Novel Coronavirus Pneumonia Emergency Response Epidemiology Team, 2020) with a median age of 42–59 years (Guan et al., 2020b; Huang et al., 2020; Ki and Task Force for 2019-nCoV, 2020; Li Q. et al., 2020; Prevention, 2020). The majority of patients are 50 years of age or older (Chen N. et al., 2020; Yang et al., 2020) and fewer than 1% of patients are under 10 years of age (Novel Coronavirus Pneumonia Emergency Response Epidemiology Team, 2020). According to the case analysis of 4,707 children with COVID-19 in China and the United States, it was found that the proportion of infant COVID-19 was relatively higher (accounting for 15% of the children), and 10.6% of the infant COVID-19 was seriously or critically ill, which was much higher than the average level of the child group (5.8%) (CDC COVID-19 Response Team, 2020; Dong et al., 2020). It is suggested that infants are more susceptible to COVID-19 and the illness is more serious. Although the incidence is higher in men than in women, the difference is not statistically significant (Ki and Task Force for 2019-nCoV, 2020; Novel Coronavirus Pneumonia Emergency Response Epidemiology Team, 2020). However, based on the meta-analysis of 77,932 patients, it was confirmed that the morbidity (OR = 1.12; 95% Cl = 1.01–1.25), severity (OR = 1.63; 95% Cl = 1.28–2.06), and mortality (OR = 1.71; 95% CI = 1.51–1.93) of males were significantly higher than those of females (Wei X. et al., 2020). In addition, elderly people with hypertension, asthma, diabetes, and other underlying diseases have a significantly increased risk of infection (Chen N. et al., 2020; Guan et al., 2020a; Huang et al., 2020). Studies have shown that 36.8% of patients with SARS-CoV-2 infection have underlying diseases, with the most common being hypertension (18.6%), cardiovascular disease (14.4%), and diabetes (11.9%) (Rodriguez-Morales et al., 2020). In addition, older patients (>60 years of age) with underlying diseases, such as cardiovascular disease, are more likely to develop severe illness, progressing to death, suggesting a poor prognosis (Wu and McGoogan, 2020; Yang et al., 2020). Infections aboard The Diamond Princess cruise ship revealed that all races can be infected, suggesting a lack of a racial component. People with A blood group have a significantly higher risk for SARS-CoV-2 infection compared with non-A blood groups, whereas O blood group has a significantly lower susceptibility for the infection compared with non-O blood groups (Zhao et al., 2020b). In summary, although the general population is vulnerable to SARS-CoV-2, a number of studies have shown that the population most at risk from SARS-CoV-2 infection is characterized by older men and people with underlying diseases. Children and infants (especially for female infants) have also been affected (Liu W. et al., 2020; Rodriguez-Morales et al., 2020; Wei M. et al., 2020), suggesting that people with lower immunity are more vulnerable to SARS-CoV-2, but more research and analysis of larger sample sizes are needed for confirmation.

### Transmission Dynamics: Incubation Period and Basic Reproduction Number

The average incubation period for 425 COVID-19 patients in Wuhan, China (as of January 22) was 5.2 days (95% CI: 4.1–7.0) (Li Q. et al., 2020). The average incubation period of the 8,866 nationwide cases in China (as of January 26) was similar, at 4.75 days (IQR: 3.0–7.2) (Yang et al., 2020). The median incubation period of 62 COVID-19 patients in Zhejiang, China (as of January 26) was about 4 days (Xu X. W. et al., 2020). In South Korea, the incubation period (as of January 20) has been reported to be about 3.6 days (median: 4). These reports are generally consistent with the incubation period of 1–14 days (mostly 3–7 days) announced by the Chinese Center for Disease Control and Prevention (CDC), but there are exceptions. For example, Guan et al. reported a median incubation period of 3 days, among 1,099 clinical retrospective samples nationwide (as of January 29), of which the longest was 24 days (Guan et al., 2020a). Bai et al. reported an incubation period of 19 days in the first case of asymptomatic infection in China (Bai et al., 2020). Hu et al. reported that one case of asymptomatic infection in Nanjing, China had an incubation period of 21 days (Hu et al., 2020). Although it cannot be ruled out that patients may have inaccurately self-reported their epidemiological histories, different studies were based on different methods, regions, and sample sizes. The incubation period varies, mostly between 3 and 7 days, and currently there are patients with an incubation period of over 14 days, which may be related to the amount of virus that initially entered the infected person and the general physical health of the infected person. These findings suggest that we need to constantly update our understanding of the incubation period of the virus, in order to prevent and block its spread more effectively.

Basic reproduction number (R_0_) is defined as the average number of secondary cases that would be generated by a primary case in a totally susceptible population. Based on an epidemiological analysis of 425 patients, the Chinese Center for Disease Control and Prevention (CDC) obtained a R_0_ for SARS-CoV-2 of 2.2 (Li Q. et al., 2020). Zhao et al. reported that SARS-CoV-2 has an R_0_ of 2.56 (95% CI: 2.49–2.63) (Zhao et al., 2020d). Another study used the serial intervals (SI) of MERS and SARS to estimate a range for R_0_ of 2.24 (95% CI: 1.96–2.55) to 3.58 (95% CI: 2.89–4.39) (Zhao et al., 2020c). Li et al. found, through an independent mathematical modeling study, that the R_0_ is about 3.39, and further reported the R_0_ before and after the Wuhan lockdown as 4.38 and 3.41, respectively (Li J. et al., 2020). These reports are generally consistent with WHO estimates of the R_0_ being between 1.4 and 2.5 (Mahase, 2020). However, Sanche et al. reported that SARS-CoV-2 has a higher median R_0_ value of 5.7 (95% CI 3.8–8.9) (Sanche et al., 2020). Although different studies have found different R_0_ values, based on different methods, regions, and sample sizes, they all suggest that SARS-CoV-2 has a strong ability to spread.

### Is Severe Illness More Likely With Increased Time Between Onset and Initial Diagnosis?

The average time between onset and initial diagnosis of the 425 confirmed patients in Wuhan, China, was 5.8 days (onset before January 1) or 4.6 days (onset after January 1) (Li Q. et al., 2020), which is generally consistent with the median time between appearance of symptoms and first consultation of 5 days (2–9 days) for the 8,866 cases nationwide, in China (Yang et al., 2020). Further analysis showed that the time between onset of severe illness and initial diagnosis was 8 days, which was significantly higher than that of patients with mild cases and patients without pneumonia, and this interval was longer in patients who died (average: 9.5 days) compared to patients who survived (average: 9 days) (Li Q. et al., 2020; Yang et al., 2020). However, asymptomatic patients or patients with mild cases have not necessarily been able to see a doctor immediately because their symptoms were not obvious, which resulted in longer intervals. Moreover, interval between onset to diagnosis may also be biased by time taken for seeking care by the patient. Therefore, the longer the time between onset and consultation, the more likely it is for severe illness to develop. More clinical samples are needed, for retrospective research, in order to draw more reliable conclusions.

## Clinical Characteristics

**Figure 2:**
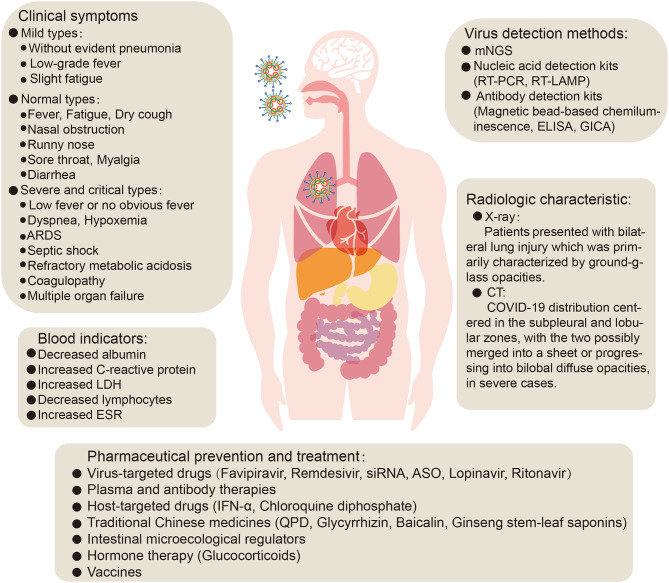
The clinical symptoms, treatment and prevention of COVID-19 pneumonia. ARDS, acute respiratory distress syndrome; LDH, lactate dehydrogenase; ESR, erythrocyte sedimentation rate; mNGS, metagenomic next-generation sequencing; RT-PCR, reverse transcription- polymerase chain reaction; RT-LAMP, reverse transcription loop-mediated isothermal amplification; ELISA, enzyme-linked immunosorbent assay; GICA, gold immunochromatography assay; siRNA, small interfering RNA; ASO, antisense oligonucleotides; IFN- α, Interferon-α; QPD, *qingfei paidu* decoction.

The typical clinical symptoms of COVID-19 are fever, fatigue, and dry cough. Atypical clinical symptoms include expectoration, headache, hemoptysis, nausea, vomiting, and diarrhea. Chemosensory dysfunction, such as loss of smell and taste, is also closely associated with COVID-19 infection but is usually recovered within 2 to 4 weeks after infection (Yan et al., 2020). Some confirmed patients are asymptomatic (Chang et al., 2020; Ki and Task Force for 2019-nCoV, 2020; Rothe et al., 2020) or have low fever, mild fatigue, or other symptoms, without presenting with pneumonia, and most recovered after 1 week (Prevention, 2020). A meta-analysis of a number of research studies was conducted, and the following abnormalities in blood indicators were found: decreased albumin (75.8%), increased C-reactive protein (58.3%), increased lactate dehydrogenase (LDH) (57.0%), decreased lymphocytes (43.1%), and increased erythrocyte sedimentation rate (ESR) (41.8%). In addition, chest X-ray examination revealed that most novel coronavirus pneumonia patients presented with bilateral lung injury (72.9%) which was primarily characterized by ground-glass opacities (68.5%) (Rodriguez-Morales et al., 2020). CT imaging analysis of 130 COVID-19 patients showed that their distribution centered in the subpleural and lobular zones, with the two possibly merged into a sheet or progressing into bilobal diffuse opacities, in severe cases (Figure 2). During the recovery period, the margins of consolidation opacities contract, the bronchi expand, and subpleural linear or fibrous opacities are the primary features (Wu J. et al., 2020). In addition, lung lesions in recovered coronavirus pneumonia patients disappear completely on CT, and there are no symptoms of fibrosis, which differs completely from SARS. Therefore, one tentative suggestion is that alveolar epithelial cells may become functional lesions.

The clinical classification of COVID-19 is primarily divided into mild, normal, severe and critical, based on clinical symptoms, clinical indicators, and imaging (Kenneson and Cannon, 2007; General Office of National Health Commission, 2020b). An analysis of the clinical typing of 1,099 confirmed patients found that the proportion of severe patients was 15.7% (Guan et al., 2020a). Classification of the 8,866 patients in China found that the proportions of severe, normal, and mild cases were 25.5, 69.9, and 4.5%, respectively (Yang et al., 2020). In addition, a study reported 18.5% critically ill patients among 72,314 patients (Novel Coronavirus Pneumonia Emergency Response Epidemiology Team, 2020). In summary, most COVID-19 patients are of the normal and mild types. Analysis of clinical characteristics showed that critically ill patients presented with moderate to low fever and even no obvious fever, in some cases, with dyspnea presenting after 1 week. In severe cases, they progressed rapidly to acute respiratory distress syndrome (ARDS), septic shock, metabolic acidosis which was difficult to correct, and coagulopathy (Prevention, 2020), as well as injury to the kidney, heart, and other organs, and even multiple organ failure (Huang et al., 2020; Wang D. et al., 2020). These clinical symptoms suggest that SARS-CoV-2 infection, in addition to affecting the lungs, also has clinical presentations that involve invasion of other organs such as liver, kidney, heart, esophagus, bladder, ileum, and pancreas (Chen N. et al., 2020; Liu F. et al., 2020; Novel Coronavirus Pneumonia Emergency Response Epidemiology Team, 2020; Xu et al., 2020b; Zou X. et al., 2020). Recent reports suggested that human liver ductal organoids were permissive to SARS-CoV-2 infection and support robust replication, which impaired the barrier and bile acid transporting functions of cholangiocytes, indicated a potential cause of liver damage by viral infection (Zhao et al., 2020a). However, liver damage in patients with SARS-CoV-2 infection may not be directly caused by viral infection, but by the systemic inflammatory response caused by therapeutic drugs or pneumonia (Chai et al., 2020). In addition, studies have confirmed that renal insufficiency is common in patients with COVID-19, which may be one of the main causes of COVID-19 eventually leading to multiple organ failure and even death (Li Z. et al., 2020). However, Xu et al. reported that, among 62 patients with SARS-CoV-2 infection in Zhejiang, kidney damage was rare (Xu X. W. et al., 2020). This may be due to factors such as the timely admission of diagnosed patients, small sample size, or the virulence of the virus may decrease with increasing passage number. In addition, the results of different analyses of the clinical characteristics of COVID-19 by different researchers are inconsistent, and may be affected by factors such as the region from which samples originated, sample size, methods of analysis, and the level of expertise in the local medical center. By comparing the sex-related hormones between 81 men of childbearing age and 100 men infected with novel coronavirus, it was found that serum luteinizing hormone (LH) increased significantly, but the ratio of testosterone (T) to LH and the ratio of male follicle stimulating hormone (FSH) to LH decreased significantly (Ma L. et al., 2020). Moreover, one study reported that ACE2 is highly expressed in renal tubular cells, leydig cells, and cells in seminiferous ducts in testis. Therefore, virus might directly bind to such ACE2 positive cells and damage the kidney and testicular tissue of patients (Fan C. et al., 2020). Shastri et al. reported that male subjects have delayed viral clearance of SARS-CoV2 than female subjects (Shastri et al., 2020). Taken together, these suggest that there is potential hypogonadism and attention should be paid to the effect of SARS-CoV-2 on the reproductive system. However, there are reports that the semen samples or testicular biopsy samples of 13 COVID-19 patients (12 recovered patients and 1 deceased) were all negative for SARS-CoV-2, suggesting that SARS-CoV-2 may not infect human reproductive system (Song et al., 2020). Therefore, further research is warranted to explore whether SARS-CoV-2 will influence the reproductive system. In addition, the pathological anatomy of patients with severe cases included bilateral diffuse alveolar injury and pulmonary interstitial mononuclear cell infiltration, with lymphocytes predominating (Xu et al., 2020b). In which case, type IV hypersensitivity may be involved in lung damage in patients with severe novel coronavirus pneumonia. In addition, at early stages of the disease, it is possible that dysfunctional antiviral IFN in type II alveolar epithelial cells causes type I hypersensitivity-like changes (complement mediated cell lysis fragments), leading to increased pulmonary exudation. In summary, early identification and timely treatment of critical cases, timely attention to the functions of various organs, and effective intervention are essential to prevent multiple organ failure and thus reduce mortality.

## Virus Detection Methods

Current detection methods for the SARS-CoV-2 virus include nucleic acid-based metagenomic next-generation sequencing (mNGS), real-time reverse transcription- polymerase chain reaction (RT-PCR), reverse transcription loop-mediated isothermal amplification (RT-LAMP) (Bhadra et al., 2015; Gu et al., 2019; Wang et al., 2019; Corman et al., 2020), and antibody detection kits based on SARS-CoV-2 antibodies in human serum or plasma (General Office of National Health Commission, 2020c; National Medical Products Administration, 2020b; Xinhua, 2020a).

The earliest technology used to test and confirm that SARS-CoV-2 is the virus infecting COVID-19 patients was mNGS (Lu et al., 2020; Ren et al., 2020). This method has high sensitivity and specificity. However, there are also many challenges, the most serious of which include high cost, long testing turnaround (about 20 h), and sequencing errors (Xuan et al., 2013; Hou et al., 2020). RT-PCR can be used to detect SARS-CoV-2 in nasopharyngeal swabs, sputum, and other lower respiratory tract secretions, blood, and feces; it is still one of the main techniques for detecting the SARS-CoV-2 virus (General Office of National Health Commission, 2020b; Jin Y. H. et al., 2020). Corman et al. further confirmed the high sensitivity and specificity of RT-PCR technology (Corman et al., 2020). However, this technique requires expensive equipment and specially trained personnel (Lamb et al., 2020) and is time-consuming (about 2–3 h or more). Improving the nucleic acid extraction and amplification process and shortening the overall testing times are urgent problems to be solved. In addition, problems such as false negatives are difficult to avoid, and it may require multiple tests to determine the status of infection. To this end, Gootenberg et al. developed a new method based on CRISPR/Cas13-based SHERLOCK technology (Gootenberg et al., 2017) for SARS-CoV-2 testing. Further studies have improved the sensitivity of novel coronavirus testing, using SHERLOCK technology, to 10–100 copies /μl, and the test can be completed within 1 h (Feng et al., 2020). However, this technology has not been validated using novel coronavirus patient samples, and therefore, it cannot be used for clinical testing. Hou et al. designed and developed a detection technology (CRISPR-nCoV) based on CRISPR (clustered regularly interspaced short palindromic repeats) and isothermal analysis to detect SARS-CoV-2; Compared to RT-PCR and mNGS, CRISPR-nCoV has a detection time as short as 40 min, while also having sensitivity and specificity comparable to mNGS (Hou et al., 2020). In addition, a reverse transcription loop-mediated isothermal amplification (RT-LAMP) method has been developed for SARS-CoV-2 testing (Lamb et al., 2020). This method reduces detection time to less than 30 min, has a low cost, and works at various pH (potential of hydrogen) and temperature ranges, while also ensuring high specificity and sensitivity (Francois et al., 2011; Lamb et al., 2020). However, this method also has drawbacks. For example, compared to RT-PCR, RT-LAMP has a higher false positive rate and cannot be used for quantitative detection (Becherer et al., 2020). At present, most of the nucleic acid test samples of suspected COVID-19 cases are upper respiratory tract samples (mainly pharynx swabs) (General Office of National Health Commission, 2020b). The non-standard collection method of pharynx swab can easily lead to misdiagnosis. And the samples collected from different parts of individuals will also affect the test result. Moreover, the collection process is extremely risky for medical staff. However, serological detection can make up for the deficiency of nucleic acid detection. Recent reports indicate that a novel coronavirus IgM/IgG antibody detection kit (magnetic bead-based chemiluminescence) has been successfully developed and approved for clinical application. This kit is a fast, high-throughput, low-cost, and safe testing method and has become another important testing method for diagnostic evidence and discharge criteria (General Office of National Health Commission, 2020c). In addition, a study has compared and evaluated the sensitivity and specificity of enzyme-linked immunosorbent assay (ELISA) kits and colloidal gold immunochromatography assay (GICA) kits for the detection of SARS-CoV-2 IgM/IgG antibodies. The study found that the sensitivity of combined detection of ELISA IgM and ELISA IgG was 87.3%. The sensitivity of combined detection of GICA IgM and GICA IgG was 82.4%, and the specificity of both was 100% (Xiang et al., 2020). These two serological detection methods are simple, rapid, and safe. However, antibody production takes time, and there are individual differences, which will interfere with the antibody test results. Therefore, the two detection methods should complement each other. At present, the National Medical Products Administration of China has approved 23 novel coronavirus detection products, including 15 novel coronavirus nucleic acid detection reagents and 8 antibody detection reagents (National Medical Products Administration, 2020a), which will further facilitate the effective control of COVID-19 epidemic.

## Pathological Characteristics

### Viral Gene Structure and Mutations

**Figure 3:**
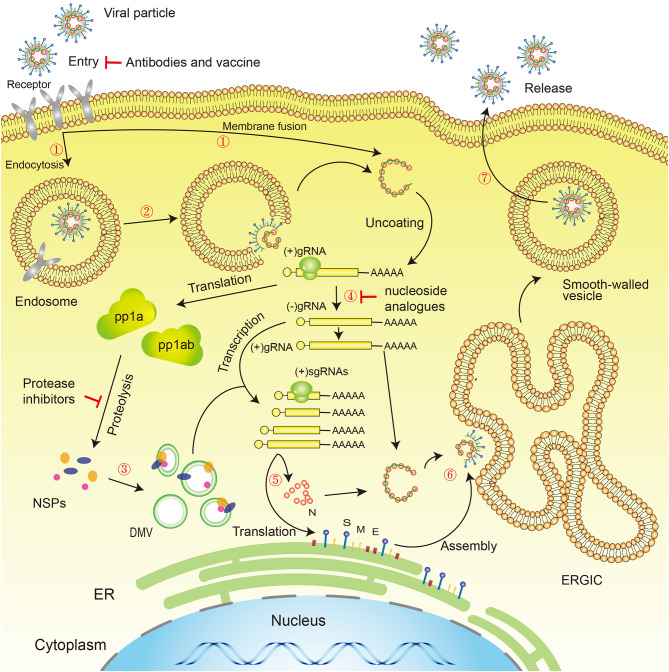
Novel coronavirus life cycle and potential drug targets. Life cycle: (1) First, the virus binds to receptors on the surface of the host cell through the S-protein and is endocytosed or directly fused with the host cell membrane into the cell; (2) Next, the lysosome degrades the lipid membrane and protein envelope on the exterior of the virus (endocytosis only); (3) Viral RNA is released into the cell, where *ORF1a* and *ORF1ab* are translated into pp1a and pp1ab, which in turn are cleaved by proteases encoded by *ORF1a* to produce multiple NSPs, forming the replication/transcription complex; (4) At the same time as the previous step, viral RNA continues to use the cell for replication; (5) The replicated viral RNA undergoes discontinuous transcription under the action of the replication/transcription complex to produce subgenomic RNA, which is translated into structural proteins in the cell's endoplasmic reticulum; (6) The resulting structural proteins assemble in the ER-Golgi intermediate compartment (ERGIC) to form the nucleocapsid and viral envelope; (7) Finally, smooth-walled vesicles containing the nascent virus particles fuse with the cell membrane, releasing the virus particles from the infected cell. Drug targets: (1) Viral S-protein; (2) 3C-like protease and papain-like protease; (3) RNA-dependent RNA polymerase (RdRP). S, Spike protein; M, Membrane protein; E, Envelope protein; N, Nucleocapsid protein; NSPs, Non-structural proteins; DMV, Double-membrane vesicles; ER, Endoplasmic reticulum; ERGIC, ER–Golgi intermediate compartment.

SARS-CoV-2 has the typical genomic characteristics of coronaviruses (CoV). It is 29,891 nucleotides in length, encodes 9,860 amino acids, and has a GC content of 38%. Sequence homology analysis shows that the similarity between SARS-CoV-2 and the SARS-like CoV isolate, bat-SL-CoVZC45, is 89.1%, and the sequence homology with SARS-CoV is 79.5% (Wu F. et al., 2020; Zhou et al., 2020). The specific replication mechanism of SARS-CoV-2 is still unclear but, as a coronavirus, SARS-CoV-2 has a form of replication similar to that of other viruses of the coronavirus family, such as SARS-CoV and MERS-CoV, in common with other virus families of the order: *Nidovirales* (Xu X. et al., 2020). SARS-CoV-2 cell entry may require two steps: the first is binding to angiotensin-converting enzyme 2 (ACE2) or CD147 or CD26 or DPP4 or TMPRSS2, and the second is cleavage of the spike protein by the Tmprss2 serine protease, which exposes the fusion peptide, allowing it to survive in low-pH endosomes (endocytosis). The virus releases RNA into the cytoplasm, initiating the process of replication in the host cell. Two-thirds of viral RNA is translated into two large polyproteins, while the rest of the viral genome is transcribed into a set of nested subgenomic mRNAs (Pasternak et al., 2006; Perlman and Netland, 2009). A variety of non-structural proteins (NSPs) are produced from the two polyproteins pp1a and pp1ab (Fehr and Perlman, 2015; Phan, 2020b), forming the viral replication/transcription complex (RTC). NSPs rearrange membranes derived from the rough endoplasmic reticulum (RER) into double membrane vesicles (DMV), within which viral replication and transcription occur, using the RTC (Knoops et al., 2008) (Figure 3).

The SARS-CoV-2 genome contains two flanking, untranslated regions (UTR) and one long open reading frame (ORF), which encodes the polyproteins. The SARS-CoV-2 genome is arranged in the order 5′ -replicase (ORF 1ab) - structural proteins [Spike (S) - Envelope (E) - Membrane (M) - Nucleocapsid (N)]−3′. SARS-CoV-2 encodes at least 27 proteins, including 15 non-structural proteins (NSP1-10, NSP12-16), 4 structural proteins (“spike protein,” “envelope protein,” “membrane protein,” and “nucleocapsid protein”), and 8 accessory proteins (ORF3a, ORF3b, ORF6, ORF7a, ORF7b, ORF8, ORF9b, and ORF14) (Ceraolo and Giorgi, 2020; Chan et al., 2020; Zhou et al., 2020). In addition, studies have compared the genomes of SARS-CoV-2 and other β-CoVs and found that the ORFs and NSPs of SARS-CoV-2 have high amino acid homology with the ORFs and NSPs of SARS-CoV (Chan et al., 2020). Studies have found that the main difference between bat SARS-like CoVs and SARS-CoV is that the spike gene of bat SARS-like CoVs has two deletions, whereas the SARS-CoV *ORF8* gene has one deletion of 29 nucleotides (Song et al., 2005; Oostra et al., 2007). Moreover, a 382-nt deletion covering almost the entire open reading frame 8 (ORF8) of SARS-CoV-2 was also observed, which may be associated with host adaptation (Su et al., 2020). These two genes have always been considered to be recombination hotspots and may become popular foci for SARS-CoV-2 research.

Like other beta coronaviruses, the SARS-CoV-2 genome has a very long *orf1ab* (Phan, 2020b) at the 5′end and a−1 frameshift between *ORF1a* and *ORF1b*, resulting in the production of two polypeptides: pp1a and pp1ab. These polypeptides are processed into NSPs by virus-encoded 3C-Lpro (chymotrypsin-like protease) or Mpro (main protease) (Ziebuhr et al., 2000; Masters, 2006; Xu et al., 2020a). Thus far, 29 missense mutations and two deletions in the gene coding for the ORF1ab polyprotein have been found (Phan, 2020a). In addition, some studies calculated the Shannon entropy, as a measure of positional variability, and made an estimate at each position of 54 SARS-CoV-2 sequences. It was found that the codon encoding serine in *ORF1ab* had silent mutations, so there may be no phenotypic differences between different virus strains (Ceraolo and Giorgi, 2020). Following *ORF1ab* are genes encoding structural proteins (Phan, 2020b). Among them, the S-protein (spike protein) encoded by the *S* gene (spike gene) plays an important role in binding to receptors on host cells, thus determining host tropism (Fung and Liu, 2019), and is also the primary target of neutralizing antibodies, from current studies (Yu F. et al., 2020). Therefore, studying mutations of the *S* gene and the structure of the SARS-CoV-2 S-protein is of great significance. A study found that three mutations (D354, Y364, and F367) are located in the receptor-binding domain of the S-protein, which may cause changes in its antigenicity (Phan, 2020a). However, there have been no studies so far on amino acid localization involved in the conformational changes of the SARS-CoV-2 S-protein. The identification of these amino acids may be of great significance. Xin et al. found an insertion of 12 bases in a gene at the junction between the coding regions for S-protein subunit S1 (spike protein1) and S2 (spike protein2). This mutation could introduce furin proteolytic sites into the S-protein, which might enhance the transmissibility of SARS-CoV-2 (Zhang et al., 2020). Wrapp et al. confirmed the structure of the SARS-CoV-2 trimer, at a resolution of 3.5 A°, in the prefusion conformation, using cryo-electron microscopy. They found that the conformation that binds to receptors had one out of the three S-protein receptor-binding domains (RBD) rotated upward (Wrapp et al., 2020). Yao et al. observed different mutations in 11 SARS-CoV-2 virus isolates from patients, including 6 different spike glycoprotein (S protein) mutations, 2 of which were single nucleotide variants (SNVs) leading to the same missense mutation (Yao H. et al., 2020). Another study found a mutant R408I from India, in the RBD domain, and the mutation may reduce the affinity of SARS-CoV-2 to ACE2 and affect the invasion of novel coronavirus to the organism (Jia et al., 2020). In addition, *ORF8* may encode a secreted protein with an α-helix and a β-sheet containing 6 strands, and there is a C or U mutation at position 28,151, which causes a serine or leucine (Ser/Leu) mutation in the encoded amino acid locus, which may affect the conformation of the peptide (Ceraolo and Giorgi, 2020; Chan et al., 2020). Moreover, 10 SARS-CoV-2 genomic sequences were obtained from bronchoalveolar lavage fluid samples of 9 patients, and their sequence homology was over 99.98% (Lu et al., 2020), indicating that the SARS-CoV-2 genomic sequence is highly conserved, which is very beneficial for whole-genome studies on this virus. These findings provide insights into pathogenesis and diagnostic optimization, and possible antiviral strategies for SARS-CoV-2, laying the foundations for vaccine development. However, 149 mutations in the novel coronavirus have already been found, evolving into two subtypes, L and S. The L subtype is more common, accounting for 70% of cases, and is more aggressive and spreads more rapidly, and has arisen relatively recently, compared to the S subtype. Further analysis shows that most patients are infected with only one of the L or S subtypes, but it cannot be ruled out that they can be infected with both (Tang et al., 2020). Recently, a study reported functional characterization of 11 patient-derived viral isolates, all of which have at least one mutation and show significant variation in cytopathic effects and viral load, up to 270-fold differences, when infecting Vero-E6 cells (Yao H. et al., 2020), which suggest that patient-derived mutations impact pathogenicity of SARS-CoV-2.

### Invasion Receptors

A series of recent studies have shown that SARS-CoV-2 can infect multiple systems of the human body, including the respiratory, cardiovascular, digestive, urogenital, and nervous system (Cai, 2020; Chai et al., 2020; Fan C. et al., 2020; Helms et al., 2020; Wang and Xu, 2020; Zou X. et al., 2020). Studies at the molecular level have revealed that SARS-CoV-2 and SARS-CoV both use the ACE2 receptor to enter cells (Zhou et al., 2020) and infect systems of the human body. Using surface plasmon resonance (SPR) and negative stain electron microscopy (EM), Wrapp et al. functionally confirmed that the affinity of SARS-CoV-2 to ACE2 is 10 to 20 times higher than that of SARS-CoV (Wrapp et al., 2020), suggesting that SARS-CoV-2 may have a higher transmissibility.

Chai et al. found that SARS-CoV-2 can bind directly to ACE2-expressing bile duct cells, leading to liver abnormalities in patients (Chai et al., 2020). Zou et al. found that the heart, esophagus, kidney, bladder, and ileum all had ACE2 expression similar to or higher than the alveoli (Zou X. et al., 2020). Fan et al. also found that ACE2 expression was most significant in the gastrointestinal tract, liver, gallbladder, kidney, bladder, and testes, suggesting that these organs may be vulnerable to SARS-CoV-2 infection (Fan C. et al., 2020). Similarly, Wang et al. used single-cell sequencing technology to evaluate the distribution and characteristics of ACE2 expression in testicular tissues of adult men, and found that ACE2 was specifically expressed in spermatogonia, Sertoli cells, and Leydig cells, suggesting that viral infection may cause disturbances in the biological function of the testes and abnormal spermatogenesis in males (Wang and Xu, 2020). Based on a public database and single-cell RNA-Seq technology, Cai (2020) found that the expression level of ACE2 in lung tissue samples of smokers was higher than in non-smokers, suggesting that the lung tissues of smokers may be more susceptible. In addition, through a larger sample size, they disproved studies reporting racial differences in the expression level of ACE2 (Zhao et al., 2020e). However, the results from the analysis of differences in ACE2 expression in the lung tissues of different populations are controversial and need to be further elucidated. Based on the China Metabolic Analytics Project (ChinaMAP) database and the 1,000 Genomes Project (1KGP) database, no ACE2 mutants, resistant to binding of the coronavirus S-protein, were found in different populations, but the diversity of genetic backgrounds among different populations and differences in mutations may affect the function of ACE2, and the expression of ACE2 may also potentially differ among different populations and races in Asia (Cao Y. et al., 2020). However, patients with SARS-CoV-2 infection primarily exhibit lesions in the lungs, despite the ACE2 receptor being widely distributed in various organs of the human body, so this connection needs to be investigated further. In addition, ACE2 is highly expressed in vascular endothelial cells, which raises the question of whether the virus can cause damage to vascular endothelial cells or impact glomerular function. Xu et al. recently reported that kidney damage is rare among novel coronavirus pneumonia patients in Zhejiang (Xu X. W. et al., 2020). It cannot be ruled out that SARS-CoV-2 also uses other receptors (CD147; CD26; DPP4; TMPRSS2) to enter different systems of the human body (Shen et al., 2017; Li Y. et al., 2020; Lukassen et al., 2020; Vankadari and Wilce, 2020; Wang K. et al., 2020). The organs with ACE2-positive cells match the organs involved with the disease, as reported in clinical studies, which raises the question of whether novel coronavirus infection causes more deaths by multiple organ dysfunction syndrome (MODS) or respiratory failure. The spike protein of SARS-CoV-2 is primed by TMPRSS2 (Hoffmann et al., 2020). ACE2 and TMPRSS2 are also highly expressed on human tongue keratinocytes. Therefore, it might be possible for the virus to reproduce in tongue epithelial cells and then enter the alveoli. If so, families might become infected by sharing chopsticks or other utensils. Also, the gene expression of *TMPRSS2* is regulated by androgens, which might be a reason why men are more susceptible to the disease.

In addition to being a receptor for SARS-CoV-2 binding, ACE2 is also involved in regulating immunity. Researchers have found that, in lung adenocarcinoma tissues with increased ACE2 expression, SARS-CoV-2 infection activates pathogenic T cells to produce GM-CSF and IL6. GM-CSF activates CD14^+^ CD16^+^ inflammatory monocytes, stimulating the production of more cytokines and eventually leading to an imbalance of the immune system. This suggests that SARS-CoV-2 may cause cytokine release syndrome (CRS) and exacerbation of pneumonia through regulation of ACE2 expression levels (Chen and Zhong, 2020). SARS-CoV-2 infection, and the CRS it induces, also upregulates the expression of the viral host cell receptor ACE2, which may further accelerate viral infection and transmission (Wang and Cheng, 2020).

### Cytokine Release Syndrome

Cytokine release syndrome is a systemic inflammatory response caused by infection, certain drugs, and other factors, which leads to a sharp increase in pro-inflammatory cytokine levels. This overreaction of the immune system causes damage to the body and is an important turning point in the transition of cases from mild to severe and from severe to critical (Hay, 2018; Shimabukuro-Vornhagen et al., 2018). Although the pathophysiological mechanisms of SARS-CoV (Wong C. K. et al., 2004; He et al., 2006) and MERS-CoV (Falzarano et al., 2013; Faure et al., 2014) are not completely clear, they are related to cytokine abnormalities, which suggests there may be a similar mechanism for SARS-CoV-2. Earlier studies have shown that increased proinflammatory cytokines (such as IL1B, IL6, IL12, interferon-γ, IP10, and MCP1) in the serum of SARS patients are associated with lung inflammation and extensive lung injury (Wong C. K. et al., 2004). Infection with MERS coronavirus can induce increased concentrations of proinflammatory cytokines (interferon-γ, tumor necrosis factor α, IL15, and IL17) (Mahallawi et al., 2018). Similarly, patients infected with SARS-CoV-2 have high levels of IL1B, interferon-γ, IP10, and MCP2, but secretion of cytokines that inhibit inflammation (such as IL4 and IL10) by T-helper-2 (Th2) cells is also increased. Further analysis found that plasma IL-2, IL-7, IL-10, GCSF, IP-10, MCP1, MIP1A, and TNF-α levels were higher in 32% of patients in intensive care units (ICU: 13 cases) than in non-ICU patients (Huang et al., 2020), suggesting that CRS may be associated with the severity of disease. However, these reports differ from those for SARS-CoV infection (Wong C. K. et al., 2004). Different mechanisms may exist, and it is unknown to what extent the disruption of immune balance is responsible for the development and progression of novel coronavirus pneumonia. Based on 99 clinical cases of SARS-CoV-2 patients in Wuhan, researchers found that virus particles spread through the respiratory mucosa and infected other cells, triggering CRS, generating a series of immune responses, and causing a decrease in immune cells, such as lymphocytes. Some patients progressed rapidly, developing ARDS, septic shock and, eventually, multiple organ failure (Chen N. et al., 2020). Lymphopenia is common in patients with novel coronavirus pneumonia, especially for T and NK cells, whereas the number of B cells does not change significantly. However, lymphocytes do not have ACE2 receptors. So, theoretically, the virus does not infect lymphocytes, and there has been no evidence of novel coronavirus infection of lymphocytes. The cause of lymphopenia is yet to be discovered. It is possible that SARS-CoV-2 acts as a superantigen, to activate T cells in large numbers, resulting in apoptosis, which in turn causes lymphopenia. An alternative explanation is that the microenvironment for lymphocyte development and differentiation is impaired, due to multiple organ failure. If peripheral blood lymphocytes are decreased, this would cause immunosuppression and might lead to secondary microbial infections or to tumors in critically ill patients. It is useful to speculate whether lymphocyte dynamics could be used as a predictor of patients becoming critically ill. At present, the mechanisms of cytokine release syndrome, and the connection with lymphocyte number, in SARS-CoV-2 infection, are speculative. In conclusion, CRS might cause patients with SARS-CoV-2 infection to transition to a serious prognosis or even death, and its pathogenesis requires further investigation. Severe COVID-19 cases may benefit from IL-6 pathway inhibition given the associated CRS- and sHLH-like serum cytokine elevations, which may be a target of the treatment of Covid-19 infected patients (Moore and June, 2020). Moreover, currently, there are also reports suggesting that cytokine receptors Fc-fusion proteins potentially serve as an antibody-like decoy to dampen the excessive cytokine levels as a strategy of the treatment of SARS-CoV-2 infected patients (Hao et al., 2020).

## Progress in Pharmaceutical Prevention and Treatment

To date, no specific antiviral therapy has been approved for the treatment of SARS-CoV-2 infection, in common with previous SARS (Avendano et al., 2003) and MERS (Zumla et al., 2015) outbreaks. Fortunately, the WHO and national governments have emphasized the development of vaccines and drugs for the prevention and treatment of infections (Mehand et al., 2018), and many drug studies are actively progressing. At present, COVID-19 patients are generally given symptomatic treatment, and supportive treatment is given, as necessary, for critically ill patients (General Office of National Health Commission, 2020b). Several potential strategies are being considered for the treatment of COVID-19 patients, including virus-targeted drugs, plasma and antibody therapies, host-targeted drugs, traditional Chinese medicines, intestinal microecological regulators, hormone therapy, and vaccines (Figures 2, 3).

### Virus-Targeted Drugs

According to genomic analysis, four enzymes expressed by SARS-CoV-2: chymotrypsin-like protease (3CLpro), papain-like protease (PLpro), helicase, and RNA-dependent RNA polymerase (RdRp), have highly conserved catalytic sites and high homology with SARS-CoV and MERS-CoV sequences. Predictions of protein structure show that the key drug-binding pockets of these enzymes are also highly conserved (Morse et al., 2020), suggesting that these enzymes would make potential targets for drug development (Tsai et al., 2006; Anderson et al., 2009).

There have been reports of approved protease inhibitors (lopinavir and ritonavir) showing activity against SARS and MERS (Zumla et al., 2016). Homology modeling methods have been used to construct structural models of two SARS-CoV-2 proteases, coronavirus endopeptidase C30 (CEP_C30) and papain like viral protease (PLVP), and it was found that CEP_C30 binds lopinavir and ritonavir more avidly, suggesting that the therapeutic effect of ritonavir and lopinavir on COVID-19 may be mainly due to their inhibitory effect on CEP_C30 (Lin et al., 2020). It remains questionable whether HIV protease inhibitors can effectively inhibit CEP_C30 and PLVP from SARS-CoV-2, *in vivo*, and exert therapeutic effects. Recently, controlled clinical trials were conducted on 134 confirmed patients with novel coronavirus pneumonia. Lopinavir and ritonavir were not found to improve symptoms or shorten the time of conversion to negative viral nucleic acids in respiratory tract specimens (Chen J. et al., 2020). Cao et al. recently also reported that no benefit was observed with lopinavir–ritonavir treatment beyond standard care in hospitalized adult patients with severe Covid-19 (Cao B. et al., 2020), so their effectiveness remains to be examined by further clinical studies.

In addition, nucleoside analogs of adenine or guanine derivatives can be used to target RNA-dependent RNA polymerase (RdRP), to block viral RNA synthesis. Favipiravir (T-705), a guanine analog used in the treatment of influenza, can effectively inhibit RdRP of RNA viruses, such as influenza virus, Ebola virus, flavivirus, chikungunya virus, norovirus, and enterovirus (De Clercq, 2019), and recent studies have reported that favipiravir has anti-SARS-CoV-2 activity (EC_50_ in Vero E6 cells = 61.88 μM) (Wang M. et al., 2020). Recently, Chinese researchers have completed clinical studies of favipiravir, which shows promising clinical efficacy in treating the novel coronavirus pneumonia. Favipiravir will be included in the treatment plan in the future within the safety, obvious efficacy and availability of the drug (Daily, 2020). Another potential treatment, remdesivir (GS-5734), is a phosphoramidate prodrug of an adenine derivative, and its chemical structure is similar to that of the HIV reverse transcriptase inhibitor, tenofovir alafenamide. Studies have shown that remdesivir can interfere with viral polymerase and it shows efficacy against MERS, in mouse models (Sheahan et al., 2020). Other studies have reported that remdesivir inhibits SARS-CoV-2 *in vitro* (EC_50_ in Vero E6 cells = 0.77 μM) (Wang M. et al., 2020). This indicates that remdesivir has broad-spectrum activity against SARS-CoV-2 and related coronaviruses (including SARS and MERS coronavirus) in cell culture and animal models (Sheahan et al., 2017; Wang M. et al., 2020). Moreover, Gao et al. recently also reported the cryo-EM structure of SARS-CoV-2 RdRP and provided a comparative analysis to show how remdesivir binds to this polymerase, which further showed the potential to treat patients in the clinic (Gao Y. et al., 2020). In addition, remdesivir has already had effective results in the United States in the fight against novel coronavirus pneumonia, in an individual case (Holshue et al., 2020). However, this is only a single case and is not sufficient to prove that remdesivir can be used to treat COVID-19 patients. Therefore, remdesivir must undergo complete clinical drug validation, and clinical trials to evaluate the effectiveness and safety of the drug for COVID-19. Unfortunately, clinical trials of redaciclovir in China have shown that its overall benefit in people with advanced infection may be small (Ed et al., 2020). In addition, through animal experiments, a team found that high doses of redaciclovir may cause testicular toxicity, resulting in a decline in sperm quality in mice (Fan J. et al., 2020). Therefore, further evaluation of the effectiveness and safety of the drug is needed.

In addition to targeting SARS-CoV-2 surface proteins, drugs can also degrade the RNA genome itself, and achieve therapeutic effects. Reports have analyzed the feasibility of using oligonucleotides to target the SARS-CoV-2 RNA genome, namely, small interfering RNAs (siRNAs) or antisense oligonucleotides (ASOs), as treatment strategies (Kruse, 2020). However, the conserved RNA sequence domain of SARS-CoV-2 is currently unknown, so effective siRNAs cannot be accurately designed and ASOs have significant limitations. The recognition of conserved sequences is essential for optimizing the siRNA targeting site and to avoid viral escape. Currently, siRNA and ASO treatment methods are produced, primarily, for rare diseases, and resources are not available to quickly manufacture drugs in this way. Recently, a team developed the lipopeptide EK1C4 based on a previous pan-coronavirus fusion inhibitor EK1 and found that EK1C4 showed strong inhibitory activity on SARS-CoV-2 S protein-mediated membrane fusion and PsV (Pseudovirus) infection. Animal experiments have found that intranasal administration of EK1C4 protects mice from infection before or after challenge with HCoV-OC43, suggesting that EK1C4 may be used to prevent and treat SARS-CoV-2 and other emerging SARSr-CoV infections that are currently circulating (Xia et al., 2020a). Another team found that the APN01, clinical grade recombinant human ACE2 protein (hrsACE2), purified *in vitro* could effectively weaken the ability of SARS-CoV-2 to infect cells in the early stage of SARS-CoV2 infection, up to 1,000–5,000 times (Monteil et al., 2020). However, this study is still limited to the level of cells and organs, which is still far from clinical application. This indicates that recombinant ACE2 may have potential value in the diagnosis, prevention, and treatment of SARS-CoV-2.

### Plasma and Antibody Therapies

Plasma therapy is a passive immunotherapy method, used in the 2003 SARS outbreak and for MERS in 2012 (Wong et al., 2003; Brown et al., 2013), and has been suggested as a treatment for COVID-19. Guo et al. found that SARS-CoV antibodies could persist at high concentrations for over 12 years, in cases that were cured after SARS infection in 2003. They also suggested that related antibodies may also have some therapeutic effect during the 2020 SARS-CoV-2 outbreak (Guo X. et al., 2020). With the current increase in the number of cured COVID-19 patients, this is also a low-technology and relatively safe therapeutic option, as long as a sufficiently high antibody titer is maintained. At present, a small number of clinical trials have found that the plasma of recovered patients has good efficacy for patients with critical novel coronavirus pneumonia (Duan et al., 2020; Xinhua, 2020b), and a study has also found that in a preliminary uncontrolled case studies of 5 patients with severe COVID-19, the rehabilitation with plasma containing neutralizing antibodies can improve their clinical status (Shen et al., 2020), but this treatment also suffers from ethical and sourcing problems. It is difficult to promote widely, in the short term, due to the lack of large-sample validation, randomized controlled trials, and well-designed clinical trials (Mair-Jenkins et al., 2015; Marano et al., 2016), so the widespread use of plasma therapy is some distance away.

With respect to antibodies, research on antibodies against S-protein (spike protein) is currently a popular topic. For example, using previous anti-SARS drugs, Tian et al. confirmed that the SARS-CoV-specific human monoclonal antibody CR3022 can effectively bind the receptor-binding domain (RBD) of SARS-CoV-2. In addition, the monoclonal antibody epitope does not overlap with the ACE2 binding site in the SARS-CoV-2 RBD, indicating that the monoclonal antibody should neutralize the virus and prevent the virus from binding to human cell receptor proteins such as ACE2, which may allow it to exert a preventive and therapeutic role (Tian et al., 2020). Research on antibodies against the ACE2 receptor (Lu et al., 2020; Xu X. et al., 2020) of SARS-CoV-2 is also a promising subject. In a study by Lei et al., the extracellular domain of human ACE2 was linked to the Fc region of human immunoglobulin IgG1, to produce a new recombinant protein with high affinity to the receptor binding domains (RBD) of SARS-CoV and SARS-CoV-2, as well as having the required pharmacological properties. Meanwhile, this fusion protein effectively neutralized SARS-CoV and SARS-CoV-2 viruses *in vitro* (Lei et al., 2020). However, a note of caution with respect to antibody therapy: the specific antibodies induced may also be involved in the pathogenesis of critically ill patients, in addition to neutralizing and blocking viral infection (Liu L. et al., 2019). Nevertheless, monoclonal antibodies generally have more specific drug targets than small-molecule drugs, so they have fewer toxic side effects. However, due to the long development cycle of monoclonal antibodies, they will arrive relatively late for clinical application. It is believed that experience in the development of SARS monoclonal antibodies, or new applications of old drugs, may accelerate the development of SARS-CoV-2 monoclonal antibody treatments.

### Host-Targeted Drugs

Interferon-α (IFN- α) inhibits animal and human coronavirus replication (Turner et al., 1986; Pei et al., 2001). For the current novel coronavirus, clinical guidelines recommend IFN-α (5,000,000 U) as an antiviral treatment (General Office of National Health Commission, 2020b). In addition, chloroquine diphosphate has been reported as a potential broad-spectrum antiviral drug (Savarino et al., 2006; Yan et al., 2013), as it can block viral infection by increasing the endosomal pH (potential of hydrogen) required for virus-cell fusion and interfere with SARS-CoV cell receptor glycosylation (Vincent et al., 2005). Currently, a multi-center clinical trial of chloroquine diphosphate is underway in China, where it has shown significant efficacy and acceptable safety in the treatment of COVID-19. It has been reported that chloroquine has been successfully used to treat more than 100 cases of COVID-19, in China, which can improve the results of radiological examination, enhance the virus clearance rate and slow down the disease progression (Gao J. et al., 2020). However, a study has pointed out the potential dangers of the antimalarial drug chloroquine, which could lead to sudden cardiac death in patients (John et al., 2020). Another study pointed out that for critically ill patients with new coronavirus, higher doses of chloroquine diphosphate should not be recommended because of its potential safety risks, especially when taken concurrently with azithromycin and oseltamivir (Multicenter collaboration group of Department of Science Technology of Guangdong Province Health Commission of Guangdong Province for chloroquine in the treatment of novel coronavirus pneumonia, 2019; Borba et al., 2020). From this, it appears that treatment of novel coronavirus pneumonia with chloroquine diphosphate is a possibility, but further clinical trials are needed to verify its effectiveness and safety. Moreover, Xiong et al. recently reported that both their self-designed candidates (two potent inhibitors of DHODH, S312 and S416) and old drugs (Leflunomide/Teriflunomide) with dual actions of antiviral and immuno-repression may have clinical potentials not only to influenza but also to COVID-19 circulating worldwide, no matter such viruses mutate or not (Xiong et al., 2020). Moreover, MTHFD1 (the C-1-tetrahydrofolate synthase gene) inhibitor carolacton potently blocked replication of several RNA viruses including SARS-CoV-2, which would be another potential target for developing broad spectrum antiviral therapy (Anderson et al., 2020). It is also worth mentioning that angiotensin-converting enzyme inhibitors (ACEI), such as captopril, enalapril, and perindopril, used to treat hypertension and heart disease, only have an inhibitory effect on ACE activity, not ACE2. They do not inhibit ACE2, but they increase its concentration. This may have the effect of accelerating SARS-CoV-2 viral replication or cell entry, which may be one of the reasons for the relatively high mortality reported in patients with novel coronavirus pneumonia and hypertension (Fang et al., 2020). However, another study retrospectively analyzed 511 patients with COVID-19 with hypertension in multiple centers, and found that patients over 65 years of age who took Angiotensin Receptor Blocker (ARB, an antihypertensive drug) were less ill, had a lower severity of illness, and had acute respiratory failure compared with those who did not take the drug (Liu Y. et al., 2020). A team also has confirmed through a large sample of clinical studies that the benefits of using ACEIs/ARBs outweigh the risks for COVID-19 patients with indications of drugs, such as high blood pressure (Liu P. P. et al., 2020). However, a large-scale retrospective study is still needed to change the future guidelines for the application of antihypertensive drugs in patients with COVID-19 susceptibility.

### Traditional Chinese Medicines

Clinical guidelines recommend the use of traditional Chinese medicine (TCM) for the treatment of SARS-CoV-2. Clinicians have used different TCM prescriptions and proprietary Chinese medicines at different stages of the clinical treatment period, for diagnosed patients, based on the principle of TCM syndrome differentiation (General Office of National Health Commission, 2020b; Jin Y. H. et al., 2020). *Qingfei paidu* decoction (QPD) has been promoted as a general prescription in the treatment plan of COVID-19 in China (General Office of National Health Commission, 2020b). A team of researchers found that the first five main active ingredients of QPD are Quercetin, Luteolin, Kaempferol, Naringenin, and Isorhamneine, and its main purpose is to suppress inflammation, regulate immune function, and reduce lung injury by regulating multiple targets and signaling pathways, so as to achieve the purpose of treating COVID-19 (Xu D. et al., 2020). In China, there are reports that out of the 701 confirmed cases treated with Qingre Jiedu Tang, 130 were cured and discharged, 51 were relieved from clinical symptoms, 268 had improved symptoms, and 212 had no worsening symptoms (Press Conference of the Joint Prevention Control, 2020). Moreover, glycyrrhizin is the active ingredient in the traditional Chinese medicine gan cao (radix glycyrrhizae or licorice root, from the plant Glycyrrhiza glabra). Cinatl et al. found that glycyrrhizin could inhibit SARS-associated virus replication *in vitro*, and it has been used as an alternative treatment for SARS (Cinatl et al., 2003). Baicalin, a flavonoid compound isolated from huangqin (Chinese skullcap, *Scutellaria baicalensis Georgi*), also inhibits SARS coronavirus *in vitro* (Chen et al., 2004). Ginseng stem-leaf saponins (from *Panax ginseng)* can significantly enhance the specific antibody response to Newcastle disease virus and infectious bronchitis virus (Ma et al., 2019). Traditional Chinese medicine is considered as an option for enhancing host immunity against SARS-CoV-2 infection. Currently, TCM treatments have shown preliminary success, and about 15 clinical trials have been registered in China (Maxmen, 2020).

### Intestinal Tract Microecological Regulators

Some articles point out that a very large proportion of COVID-19 patients who initially present atypically, have gastrointestinal symptoms (Gao Q. Y. et al., 2020). Studies have found that ACE2 mRNA is highly expressed in the small intestine of healthy individuals. Further analysis found that exposure of proximal and distal small intestinal epithelial cells to foreign pathogens significantly increased ACE2 expression (Liang et al., 2020). Mutations in the ACE2 receptor may reduce expression of antibacterial peptides in intestinal cells and cause changes in intestinal microecology (Hashimoto et al., 2012). Therefore, researchers speculated that COVID-19 may affect the intestinal flora via the ACE2 receptor (Gao Q. Y. et al., 2020). Previous studies have shown that regulation of the intestinal flora can reduce enteritis and respiratory-associated lung infection and can reverse certain side effects of antibiotics, thereby preventing the early replication of influenza virus in lung epithelial cells (Bradley et al., 2019). Therefore, intestinal tract microecological regulators can be used in the treatment of severe and critical cases, to maintain intestinal microbial balance and prevent secondary bacterial infections (General Office of National Health Commission, 2020a). Although there is no direct clinical evidence that regulating intestinal flora can play a role in the treatment of COVID-19, targeting the intestinal flora is still a potential treatment option, or at least as an adjuvant treatment (Gao Q. Y. et al., 2020).

### Hormonal Treatment

It is still controversial whether treatment of COVID-19 with glucocorticoids results in ARDS. Studies have found that glucocorticoids increase mortality risk in influenza patients and also delay virus clearance in patients infected with MERS coronavirus. Although glucocorticoids have been widely used to treat SARS, there is insufficient evidence demonstrating benefit to patients, and instead there is clear evidence suggesting short- and long-term adverse effects (Russell et al., 2020). Therefore, with the exception of patients with acute exacerbation of chronic obstructive pulmonary disease and other indications, the United States CDC does not recommend treating COVID-19 pneumonia patients with glucocorticoids [(National Center for Immunization and Respiratory Diseases, 2020)].

### Vaccines

The ultimate measure for SARS-CoV-2 epidemic control and prevention will be the use of protective vaccines. Several previous vaccination strategies for SARS-CoV, such as inactivated viruses, live attenuated viruses, viral vectors, subunit vaccines, recombinant proteins, and DNA vaccines, have been developed and tested in animals (Roper and Rehm, 2009; Graham et al., 2013), and the development of a vaccine is imminent. A similar approach has also been used in the development of experimental MERS-CoV vaccines (Du and Jiang, 2015). Escriou et al. developed a candidate vaccine (SARS-CoV-S vaccine) using a recombinant, live, attenuated measles vaccine, expressing the membrane-anchored SARS-CoV spike (S) protein and found that it could induce the highest titers of neutralizing antibodies and fully protected immunized animals from intranasal infectious challenge with SARS-CoV (Escriou et al., 2014). A study by Bodmer et al. showed that two live, attenuated measles vaccines expressing MERS-CoV S- and N-proteins could induce a strong multifunctional T cell response in a mouse model (Bodmer et al., 2018). Because SARS-CoV-2 has high homology with SARS-CoV and MERS-CoV (Morse et al., 2020), the research and development of novel coronavirus vaccines can draw from the methods of SARS and MERS vaccine development. Researchers identified SARS-CoV-derived B and T cell epitopes, through a SARS-CoV immunogenic structural protein screening study. They identified B and T cell epitopes with the same S- and N-proteins as SARS-CoV-2. Immune targeting of these epitopes may help guide the development of a vaccine against SARS-CoV-2 (Ahmed et al., 2020). In another study, two types of mRNA vaccine were designed to target virus-like particles (VLPs) and receptor-binding domain of the spike protein (S-RBD), respectively. After extensive optimization, an mRNA cocktail containing three genes was used to produce a candidate vaccine, comprising SARS-CoV-2 virus-like particles which are highly similar to natural SARS-CoV-2, but this has not yet been tested in animals. Meanwhile, another candidate vaccine expressing S-RBD mRNA is being tested for immunogenicity in mice (Xia S. et al., 2020). In addition, a candidate vaccine sensitive to MERS-CoV has been designed. It uses a harmless parainfluenza virus 5 (PIV5) to deliver the S protein of MERS-CoV to cells to produce an immune response, which provides a new strategy for the development of vaccines for SARS-CoV-2 (Li K. et al., 2020). Since the novel coronavirus is an emerging pathogen, vaccine development is expected to be difficult and to have a relatively long cycle. Currently, researchers in China have been conducting simultaneous studies along multiple technical routes, including inactivated vaccines, mRNA vaccines, recombinant protein vaccines, viral vector vaccines, DNA vaccines, and so on, and some types of vaccine have entered the animal testing stage or human trials (Chinadaily com.cn., 2020; Jiang, 2020). It's also worth pointing out that a team has demonstrated the safety and effectiveness of a purified SARS-CoV-2 virus candidate inactivated vaccine (PicovAcc) used in a rhesus monkey model, and phase I clinical trials of the vaccine have begun (Gao Q. et al., 2020). However, study has found that SARS-CoV-2 exists for a long time (two cases for up to 50 days) in COVID-19 patients who produce specific antibodies, and the production of antibodies does not mean the rapid clearance of SARS-CoV-2 (Wang B. et al., 2020). Therefore, herd immunity whether is a correct way to prevent novel coronavirus requires further research. Specific antibodies can block virus infection, however, antibody dependent enhancement (ADE), in turn, promotes infection (Tetro, 2020). Moreover, the existence of variation of viral antigens and the phenomena of immune suppression, suggested that SARS-CoV-2 vaccines are difficult to develop and need much focus. In spite of this, it is believed that a SARS-CoV-2 vaccine will be available in future.

## Outlook

The COVID-19 outbreak poses a threat to the health and lives of people worldwide. However, knowledge about the novel coronavirus remains limited. Although the world is working hard to understand COVID-19, many unknowns remain, including: (1) The phenomenon of “reversion” in COVID-19 patients, after recovery (Lan et al., 2020), the proportion estimated to be 1–14%. Infected individuals showing reversion generally had no obvious symptoms after being discharged from the hospital and were only tested positive using RT-PCR. There is no epidemiological data on whether these so-called “reversion infections” are still contagious. There is no laboratory sequencing data available to determine whether infected individuals with reversion are just “glowing embers” or a full-blown re-infection. However, the latest reports suggest that reinfection could not occur in SARS-CoV-2 infected rhesus macaques, which indicate that the primary SARS-CoV-2 infection could protect from subsequent exposures and the re-positivity from discharged patients could not be due to reinfection (Bao et al., 2020). However, Yao et al. reported that SARS-CoV-2 was remaining in pneumocytes and caused pathological changes in the lungs for a patient tested negative for consecutively three times by nasopharyngeal swab—PCR test (Yao X. -H. et al., 2020). These results suggested that more complicated issues need to be considered to find out the causes. (2) No immunological characteristics of asymptomatic infected persons have been reported. (3) Since the RRAR enzyme cleavage site is more conducive to furin cleavage of the S-protein, it has been suggested that it is harder for HIV-infected people to contract novel coronavirus. In dealing with a new virus, we need more clinical immunological evidence of how the adaptive immune system responds to it. Over the years, research on coronaviruses has produced a variety of strategies for diagnosis, prevention, and treatment. These results are likely to apply to SARS-CoV-2 or any other emerging coronavirus in the future. With continued efforts to prevent the global spread of SARS-CoV-2, we hope the pandemic will subside in a few months, in a similar way to SARS and MERS. Nevertheless, this outbreak underscores the urgent need to develop broad-spectrum antiviral drugs to fight coronaviruses. Our immediate action must be to implement infection control measures, to prevent further transmission of SARS-CoV-2.

## Author Contributions

GG, LY, and XX planned the work. GG, LY, KP, and YC drafted the manuscript. GG, KP, YC, and XX revised the manuscript. DX, KY, ZC, ND, WL, HH, LZ, and XL participated in the literature search and discussion. KP and HH arranged figures. All authors read and approved the final manuscript.

## Conflict of Interest

The authors declare that the research was conducted in the absence of any commercial or financial relationships that could be construed as a potential conflict of interest.
